# Pain relief in labour: a qualitative study to determine how to support women to make decisions about pain relief in labour

**DOI:** 10.1186/1471-2393-14-6

**Published:** 2014-01-08

**Authors:** Joanne E Lally, Richard G Thomson, Sheila MacPhail, Catherine Exley

**Affiliations:** 1 Institute of Health and Society, Baddiley Clark Building, Newcastle University, Baddiley-Clark Building Richardson Road, Newcastle upon Tyne NE2 4AX, United Kingdom; 2 Newcastle Upon Tyne NHS Foundation Hospitals Trust, Royal Victoria Infirmary, Queen Victoria Road, Newcastle upon Tyne NE1 4LP, United Kingdom

**Keywords:** Pain relief, Shared decision making, Antenatal education, Risk communication

## Abstract

**Background:**

Engagement in decision making is a key priority of modern healthcare. Women are encouraged to make decisions about pain relief in labour in the ante-natal period based upon their expectations of what labour pain will be like. Many women find this planning difficult. The aim of this qualitative study was to explore how women can be better supported in preparing for, and making, decisions during pregnancy and labour regarding pain management.

**Methods:**

Semi-structured interviews were conducted with 13 primiparous and 10 multiparous women at 36 weeks of pregnancy and again within six weeks postnatally. Data collection and analysis occurred concurrently to identify key themes.

**Results:**

Three main themes emerged from the data. Firstly, during pregnancy women expressed a degree of uncertainty about the level of pain they would experience in labour and the effect of different methods of pain relief. Secondly, women reflected on how decisions had been made regarding pain management in labour and the degree to which they had felt comfortable making these decisions. Finally, women discussed their perceived levels of control, both desired and experienced, over both their bodies and the decisions they were making.

**Conclusion:**

This study suggests that the current approach of antenatal preparation in the NHS, of asking women to make decisions antenatally for pain relief in labour, needs reviewing. It would be more beneficial to concentrate efforts on better informing women and on engaging them in discussions around their values, expectations and preferences and how these affect each specific choice rather than expecting them to make to make firm decisions in advance of such an unpredictable event as labour.

## Background

One of the key priorities of modern healthcare is to encourage engagement in decision making [1], with an acknowledged right for all patients to have an active role in decisions that affect their healthcare [2,3]. The National Patient Survey [1] asks patients if they were as involved as they wanted to be in decisions about their healthcare, a question which gives an indication of the importance of decision making in today’s healthcare. That survey found that 48% of inpatients following discharge expressed a desire for greater involvement in decisions about their care, a statistic that has not changed over recent years [1].

Understanding what a patient expects of their interactions with healthcare professionals and what involvement they want in decisions about healthcare, often referred to as health expectations, is becoming increasingly important [4]. Efforts have been made to support patients to develop realistic expectations and enable them to make treatment decisions that are in line with these expectations therefore reducing any decisional conflict patients may experience [5]. A patient’s expectations about their treatment, experience and outcomes are important in most decisions, but especially so in relation to pain relief in labour [6]. As well as considering health expectations generally, health professionals who care for pregnant women have the additional challenge of ensuring they provide support to women to shape their expectations within a framework that is achievable, while at the same time ensuring they support them adequately with their labour and birth [7].

Health expectations can be defined as an individual’s assessment of their most likely outcomes [7], as well as the treatment and care they will encounter. A patient’s health expectations are formed from accessing multiple information sources, combined with the perceived severity of the symptoms, the patient’s perceived vulnerability, alongside any previous experience and knowledge [8]. Thompson and Sunol [9] stated that there are four different types of expectations: the first type is ‘ideal’, referring to a person’s desired or preferred outcomes; the second is ‘predicted’, which relates to what a person actually expects; the third type is ‘normative’, which is what should happen; and the final type is ‘unformed’, where a person has yet to consider the options, outcomes or form any expectations. When healthcare professionals discuss supporting women in developing their expectations for pain in labour, there needs to be clarity about which particular expectations they are referring to.

Many of the decisions women make during pregnancy about how they wish to manage their pain in labour are based on what they expect labour and labour pain to be like [10]. According to Thompson and Sunol’s definition of expectations, this relates to a woman’s predicted expectation [9]. In order to form these antenatal expectations, women combine information they receive from a variety of sources [11] with other beliefs they have regarding pain in labour. Women consider how painful they expect labour to be, how effective and acceptable the various forms of pain relief are, and their inherent knowledge [12], along with how they think they will cope with the pain. Pregnant women’s expectations continually change throughout pregnancy as they receive and review new information [13]. Expectations formed by pregnant women include how painful they think labour might be [14]; whether they are expecting labour pain to be a positive [15,16] or negative experience [17]; what relief they perceive they will get from the various methods of pain management [18,19]; and how long labour will last [20]. In contrast to the pain of an illness, pain associated with labour and birth, though intense, has been said to imply a clear purpose [21]; it is a normal part of labour [22] and ought to be treated accordingly. Some women express hopes of what might happen, equating to ‘an ideal expectation, a person’s assessment of their most desirable outcome’ [7]. For example, a recent review identified a distinction between women’s hope for a drug free labour and, at the same time, their understanding and expectation that they may find that they need some sort of pain relief [10].

The aim of this paper is to draw upon interviews with women to explore how they can be better supported in preparing for, and making, decisions during pregnancy and labour regarding pain relief. The key objectives were to explore how women’s hopes and expectations regarding pain and its relief in labour are formed during pregnancy, to explore how and when decisions are made during pregnancy regarding pain relief in labour; and finally to consider how women’s expectations may influence the decisions they make.

## Method

A qualitative study was undertaken to explore women’s perceptions, expectations and experiences of childbirth and their decision making processes regarding pain relief in labour. This chosen approach enabled an interpretation of the different ways in which women make sense of their experience [23], to add to our understanding of women’s experiences in order to be able to offer them appropriate support.

Participants consisted of pregnant women (n = 32) in the north east of England. Patient information leaflets were given to women by their community midwives and written informed consent was obtained from all those pregnant women who took part in the study by the researcher (JL) before the interview was conducted. Women were recruited through two local midwifery teams, chosen because they served two very different socioeconomic groups. Purposive sampling was used to capture a range of different views and experiences e.g. age, area of residence and educational achievement [24]. Appropriate local ethics approval was gained from the Newcastle and North Tyneside Local NHS Ethics Committee.

Semi structured interviews were conducted by JL with women between 28 and 36 weeks of pregnancy in their own homes and lasted between 35 minutes and one hour. The topic guide was used to frame the questioning during the interviews to ensure key areas were discussed; the prompts were not necessarily asked sequentially in every interview, but in such a way as to allow sufficient flexibility in the interview to enable further discussion of any new issues that were raised by the women.

### Topic Guide for interviews with pregnant women

Have you thought about how you are going to manage your pain in labour yet?

What do you know about the different methods of pain relief?

Have you made any decisions about which methods of pain management you are going to use?

Where have you been getting your information from?

Are there any areas that you feel you don’t have information about that you would like?

Are there any other issues that have not been raised today that you want to raise about pain relief?

The interviewer was careful to use terms such as ‘pain management’ to enable discussion about methods of coping and relaxation, and ‘pain relief’ when trying to find out more about pharmacological and non-pharmacological methods of pain relief.

All interviews were audio recorded and transcribed verbatim. The interviews explored women’s experiences, expectations, decision-making preferences, sources of information and other resources for decision making, birth planning and pain-relief choices. All women were re-interviewed within six weeks of giving birth to enable comparison between their hopes and expectations and their actual experience of labour and pain management, and to ascertain what factors had influenced any changes in the decisions they had made.

The transcripts that comprised the formal data for analysis were coded (JL) and subsequently discussed at data sessions (JL/CE) to develop a coding frame which was then applied to subsequent interviews. The large volume of data was managed using NVivo software. Quotes from the women themselves are included throughout to illustrate interpretation. Respondents are identified after quotes by their pseudonym, their age group, whether they were multiparous or primiparous, and whether the interview was conducted antenatally or postnatally: e.g. (Jane, >30, Primiparous, Antenatal). The discussions with the women, although aided by the topic guide, were determined by the women themselves and as such this paper focuses on the issues raised by them.

## Results

The data from the interviews identified three key issues related to decision making about pain relief in labour. Firstly, during pregnancy women expressed a degree of uncertainty about the level of pain they would experience in labour and the effect of different methods of pain relief. Secondly, women reflected upon how decisions had been made regarding pain management in labour and the degree to which they had felt comfortable making these decisions. Finally, women discussed their perceived levels of control, both desired and experienced, over both their bodies and the decisions they were making.

During the antenatal interviews the women tended to focus mainly on pharmacological interventions rather than non-pharmacological alternatives, although several women did talk about the importance of breathing and relaxation and the benefits that continuous support could provide in management of their pain. Some women who attended NHS ante-natal classes reported, in their post natal interviews, that little information was given to them regarding approaches such as relaxation and breathing; consequently they did not feel as confident in using them as they would have liked.

### Level and type of pain and pain relief during labour

The way a woman views labour pain antenatally will shape the choices she makes regarding pain management as labour begins and some would argue that these antenatal expectations will shape her actual experience of labour [25]. For example, if a woman is anticipating pain in labour to be unbearable, she may already be looking at ways of alleviating that pain. The majority of women throughout this study remarked that, despite attending several different types of antenatal preparation classes, they were still uncertain about many aspects of labour; it was this uncertainty that was shaping their plans for labour.

They [antenatal educators] probably were at the point where they’re kind of talking about the pain relief options they start out about relaxation techniques and spend five minutes saying oh you know yes it can help to keep breathing and keep relaxed it will help as much as possible, and that was probably all they kind of spoke about it. They didn’t actually do anything, actual practicing sort of these techniques. (Linda, 20–30, Primiparous, Antenatal).

As Green et al. [26] found in their work, many of the women in this study wanted to avoid or minimise pharmacological pain relief, but had a lack of self-confidence about whether they could cope or use the suggested breathing and relaxation techniques; they were unsure about how painful labour would be or how long would it last. For many these doubts led to them opting at an early stage for one of the pharmacological pain relief options.

“You don’t know how painful it is going to be, it’s the fear of the unexpected” (Pauline, >30, Primip, Antenatal).

This cause for concern was exacerbated by the fact that, despite the uncertainty women had about the level of pain in labour, they were being asked during their antenatal care to make choices about which pain relief they would choose. The only strong preference many women expressed was related to either their mobility during labour or issues relating to being in control. However, women reported the difficulties of making explicit decisions prior to labour about the type of pain relief they might want during labour.

“I guess, it’s the idea of being out of control, you know out of control sort of , not, I mean this is dreadful, I’ve got no idea but just the control, just the idea of it all being controlled through drugs and things like that. And you’re not actually being able; you know, having to be told what to do”. (Lynne, >30, Primp, Antenatal).

“You see I don’t want to be told, I want to know what’s going on and try and make my own choices and decisions do you know what I mean, be in control”. (Susan, >30, Primip, Antenatal).

All women in the study were concerned to a greater or lesser degree that they felt they had little insight about what to expect of the pain in labour. However, none reported discussing these concerns with their midwives, the implication being that there was not enough time within their antenatal appointments to cover the information the women needed.

“Every time I go and see the midwife it seems to be blood pressure, urine analysis, lie on the couch, feel for the head, check the heart, see you in however many weeks’ time. So it all seems to be sort of a physical check which obviously is probably the most important thing for the delivery. I know they haven’t got the time it’s like a GP appointment, it’s all scheduled, they just don’t have the time for it really”. (Joan, <20, Primip, Antenatal).

Often time is limited, and appointments need to cover essential health checks, but women felt they would benefit from more time discussing issues that were important to them, including information on pain management and coping strategies [27], rather than a dominance of information on pharmacological methods of pain management.

### Decision making

Although some research reports that women want to be involved in decision making about labour [28], others report that some women may find the thought of being involved daunting and may wish to transfer responsibility to someone else in this matter [29] Pg.78, claiming that women’s involvement may burden them with an overwhelming sense of responsibility that they do not wish to have [25].

The data from this study do not provide any clear evidence that making decisions antenatally about specific methods of pain relief in labour was a priority. Women in this study wanted to be informed and involved in decision making, but many did not feel that deciding on specific options of pain management was a priority. Many, including Pauline, found it difficult to determine how they could actually make decisions for labour when they were still not entirely sure what to expect.

“You can get into labour and you’ll be given lots of choices by the midwives and again it’s probably not the time to be given choices. It’s kind of you… almost want someone to say well actually I think this is actually best for you and the baby now, and it is a bit worrying …you’re making decisions about the best thing to do but you don’t know, you just don’t know”. (Pauline, >30, Primp, Antenatal).

Pauline wanted someone during her labour to explain what would be the best for her baby, which has been termed by some as a decision by explanation [30]. In this situation the woman still takes part in the decision, but the midwife explains the best options available, given the stage of labour and the ability of the woman to cope with pain, and supports her in making a decision.

Women identified the importance of support from the delivery suite midwives in helping them to achieve the birth they wanted. After the birth of her second child, Jan, like the majority of women in the study, was very positive about the support she had received from midwives.

I got into the delivery room she was very, very good. She was very sort of right, what do you want? You’re doing very well, this is all, immediately the emotional support kicked in big time which was just what was needed. She sort of did a quick examination and said this is all going really well….she was just very encouraging saying this is going absolutely fine, you’re doing fantastically well (Jan, >30, Multiparous, Postnatal).

There are several ways in which women can be supported in their involvement in decision making to lessen the burden; writing a birth plan is one. A birth plan in the NHS (which may have different names in other healthcare systems) is a written document which aims to enhance involvement in the decision making process, and includes a range of options that the woman has investigated and may wish to consider at an appropriate point during labour [31,32]. The birth plan is intended to support the woman during labour by ensuring that her preferences are known by those caring for her. Brown and Lumley [32] reported that 21% of women in their study saw birth plans as a way of considering and becoming acquainted with their options, informing others of their preferences and enabling them to take part in decisions.

For some women in this study the process of writing a birth plan was a positive step to enable them to consider their options, express a preference (or a hope) and to act as an aid to them being engaged in the decision making process during labour.

“…to order my thoughts just in case I am unable to get my words out”. (Chris, >30 Multip, Antenatal).

“I want one [birth plan] in place, obviously when me notes go to them, you know, you don’t know what your pregnancy, your labour is going to turn out like second time round. So I thought I wanted something written down in the notes for when I walk in in case I maybes can’t manage to get the words out”. (Chris,>30, Multip, Antenatal).

Other women, like Gail and Lynne, felt a birth plan may actually show distrust of the healthcare professionals and that it was better to hand the decision making over to them. They decided not to write a birth plan.

“[I] completely trust the professionals, they do it three or four times a day, they know what I need”. (Lynne, >30, Primip, Antenatal).

“Just the kind of to put your, put yourself in other people’s hands a bit. Not be sort of too, you know I’m a strong independent woman, I make my own decisions… because I think, I think you’re very lucky if you can sort of get, you know, be in that situation and be able to, you know, ignore all the professional advice and just go with what you want to do and come out of it the other end, you know having a good experience. I think it’s just one of those things where other people sometimes know better than you”. (Gail, >30, Primp, Postnatal).

Generally a good relationship with caregivers is a strong predictor of a positive experience of childbirth, including feeling that caregivers involve them in decisions about their care [33]. Women in this study identified support from the midwives as key factors in their birth experience. The support received either helped them cope with the pain they were experiencing or enabled them to feel engaged in the decision making process throughout labour.

### Types of control during labour

Women in this study expressed very strong opinions about not wanting to feel out of control.

“It’s just the idea of, being out of control, just the idea of it all being controlled through drugs and things like that. And you’re not actually being able, you know having to be told what to do”. (Lynne, >30, Primip, Antenatal).

“I don’t want to be just like off it when I’m…. I don’t know. Just the thought of like not being in, like I know you’re not in control of yourself anyway, but do you know what I mean? I don’t know, just not… like I wanna be as grounded as I can be really”. (Joan, <20, Primp, Antenatal).

Research suggests that women want to remain in control in labour [34,35], although what type of control is not clear. Control for the women in this study meant a variety of things - control over what pain relief they had; control over decisions, for example about mobility; or control over their own bodies. Women had strong views as to how specific pain management choices would impact upon their control; this influenced decisions they made regarding their pain. Women in this study had a wide range of expectations as to how different forms of pharmacological pain relief would either enhance or diminish their sense of control during labour. Susan had a strong sense that she would be able to cope with the pain and remain in control during labour, and was supported in this endeavor by her midwife.

Don’t jump straight into getting pain relief give it like, give it a chance do I know what I mean? Because it’s only really at the peak of the contractions where it gets really unbearable and… just give it a chance I suppose….. One of them says to me that it’s going to end shortly, do you know what I mean? You’d be so disappointed if you go and get the epidural and that. And then I kept remembering that when I was in labour just keep going, doing what you’re doing. Trust your body I suppose. (Susan, 20–30, Primiparous, Postnatal).

Women had formed opinions about which pharmacological options of pain relief would reduce their control over their own bodies, but these views varied. Mary, for example, identified specific drugs that she was concerned would take away her ability to be in control of her pain.

“I don’t want morphine, pethidine or, or an epidural I don’t want the epidural because I like to feel in control of pain, I don’t like it taken away so that I can’t feel what’s happening”. (Mary, 20–30, Primp, Antenatal).

Some women, like Lynne, saw Entonox (nitrous oxide and oxygen) as providing pain relief whilst allowing them to remain in control, in contrast to thinking that pethidine would diminish their control.

“I guess, it [Entonox] sort of, just sort of taking the edge off, giving you something to, you know con, not concentrate on, but, but still maintaining… that you know, that you do what you think you have to do and, and it’s still in your control”. (Lynne,>30, Primip, Antenatal).

Naomi commented that her decision not to opt for pharmacological pain relief was based upon information about the lack of control it caused that she had received from her sister, rather than from her midwife (family and friends were the most referred to source of information for all of the women in the study).

“I knew about like pethidine, my sister, I think it was, she might have had diamorphine..is that right? And when she told me how hers went I just , I just knew it wasn’t for me and… like with me wanting to be in control and to know exactly what was going on and to be aware of everything”. (Naomi, 20–30, Multip, Postnatal).

When an epidural is administered, generally women cannot feel pain and are unable to move from the bed, as the baby is being continuously monitored. The women in this study did not report that these movement restrictions diminished their control. Indeed, several women, such as Naomi and Alison, saw choosing an epidural to relieve their pain as a way of preventing them from losing control or of regaining the control they had lost because of the pain they were in.

“I was chatting away [after the epidural was inserted], I was making decisions with the midwife, I mean it’s like quite big decisions at that point which I was pleased I was able to do….. if I was on gas and air I wouldn’t have got that, any of that across……..definitely. I was able to like take part take part in it and enjoy it as well”.(Naomi, 20–30, Multip, Postnatal).

“You can understand why people used to bite twigs you know in the olden days ) because I just wanted to get a hold of something. …Em, but then it went from all panic stations to me lying down watching the telly [after the epidural had been inserted] It was great, it went from mayhem to totally chilled out relaxed. …. That’s why I’m glad I done it [epidural] ‘cause the whole thing it made me calmer”. (Alison, 20-30, Primp, Postnatal)).

One woman expressed a very different view from everyone else; the only way she could envisage remaining in control for subsequent births would be to labour and deliver at home, in familiar surroundings with the continuous support of a midwife;

“I don’t want it to go like it went last time. I had quite a traumatic first birth and I had basically a series of events happened and I felt that I had lost control and I hadn’t been informed properly when I was in labour. So, things kind of went out of control. This time round I’m, I’m planning to be at home because that gives me an element of control that I didn’t feel that I had when I was in hospital”. (Rebecca, >30, Multip, Antenatal)).

Hence, control is a major factor in a woman’s overall birth experience, be this their ‘internal’ control of their behaviour or external control of aspects of labour such as decision making [35]. Women in this study referred not only to the pain affecting their control [36], but also to specific methods of pain relief having an impact on their internal control as referred to by Green and Baston [35].

## Discussion

An overarching finding identified throughout the results of this study was that women felt that they were not in a position to make decisions about their plans for pain management in advance of labour. Neither primiparous nor multiparous women were able to make decisions. They reported lacking knowledge on how to use non-pharmacological methods of pain management, such as breathing and relaxation, alongside limited knowledge on how painful labour would be and how the various forms of pain relief would affect their control. Women mainly stated that they wanted to wait and see before deciding on pain relief.

The first major issue, raised by all women, was the inability to imagine antenatally what labour pain would be like. Even women who had experienced labour pain before found it difficult to remember and describe the pain. Difficulties for women began to emerge during pregnancy when healthcare professionals encouraged them to make early decisions about pain management in labour. Women reported that it was difficult to make decisions when they didn’t know what to expect, specifically the degree of pain they would experience. They demonstrated a reluctance to make any firm decisions; many spoke of wanting to wait and see how they coped with the pain after labour began. Women in a study by Niven and Gijsbers also expressed their difficulty in making these decisions when they did not know how painful labour would be [37]. If women do not know what to expect of labour, it is debateable whether healthcare professionals should be asking them to make these decisions about pain relief before their labour actually begins. Current guidelines recommend that women are provided with information on labour, including how to cope with pain, at or around 36 weeks of pregnancy, with support to make informed choices [38]. The women in this study implied that they were being asked to make their decisions regarding management of pain at 36 weeks rather than being informed about their options. A more appropriate approach would be to ensure that all women are fully informed antenatally about their relevant pain relief options, including both coping strategies and pharmacological options as recommended by the guidelines. In that way they can begin to form preferences, but can reconsider these throughout pregnancy and make better decisions based on the pain they are actually experiencing during labour. The fact that women do not need to make decisions at 36 weeks should be clearly articulated to reduce this perceived pressure to make early choices before labour begins.

Leap and Anderson acknowledge that most women are fearful of pain in labour [39]. Giving a woman information, for example that contractions only last up to a minute and that her body can produce pain killing hormones to help her cope, may ease these worries as she approaches labour [39]. Information given to women needs to contain details on the effectiveness of all available options of pain management (both pharmacological and non-pharmacological), the risks, benefits and consequences of the options, and the appropriate points in labour for particular forms of pain relief to be administered. As well as information, women need to be aware of the emotional and physical support and the advocacy role that midwives can provide, which will help them achieve the labour and birth they want [40]. This approach would enable women to make decisions based on the knowledge they have gained during their antenatal preparation, and on the pain they are actually experiencing, with the full support of their midwife. This, however, could give rise to tension between women favouring a ‘wait and see approach’ since there is always uncertainty around preparing for labour and professionals wanting to know what strategies women intend to use to ensure they are fully prepared.

The second issue that arose relates to gaps in antenatal information provision to women about pain management options. One way women currently try to ensure they are informed about their options for labour is to attend antenatal education classes. It is recommended by the Royal College of Obstetricians and Gynaecologists that all women and their partners attended antenatal classes to help prepare them for labour [41]. Indeed many of the women in this study reported attending NHS antenatal classes, NCT classes or active birth classes as part of their own preparation for labour. However, the data from this study regarding women’s lack of understanding of pain and its management in labour imply that the way in which the information is currently being delivered in antenatal classes is not effective. An English National survey also found that only 6% of respondents found antenatal classes to be the most helpful source of information [42]. This may be due to the type of information being presented, the format and delivery, the source it comes from or the time it is delivered. Whatever the reason, the outcome is that this information is not being transferred into knowledge by the women. When asked in a large retrospective survey, only 20% of women indicated that a taught class was their preferred method of receiving information [43]. When women are given information on the options available, including the risks, benefits and consequences, it must be given in a way that is easily accessible and understandable, if women are to be adequately prepared for the pain of labour.

The third issue that was raised during the interviews related to decision making during pregnancy and labour. When people have choices to make about their healthcare generally, they base their decisions on the severity of the problem, the timeframe in which a decision has to be made, their prior experiences and knowledge, and their own values and expectations. The severity of the problem in labour is the degree of pain and the length of time labour will last, as well as the woman’s ability to cope with the pain. Some decisions about pain management during labour have to be made within a specific time frame, especially if complications are arising or there are concerns for the health of the baby. Also, some pharmacological pain relief can only be administered safely if birth is not imminent, so women’s choices are sometimes constrained by progression of labour. As has been discussed, women’s knowledge, expectations and experience play a large part in the decision making process.

Another important factor impacting on the decision making process is what is important to the woman. A woman, with support from her peers and caregivers, needs to identify a range of things that may impact upon her approach to labour [44] to assist in her decision making, including her beliefs, values and preferences [45,46]. Values, for example, may include a desire for a natural childbirth or placing importance upon a home delivery. A decision made is often referred to as a “good” decision with minimal decisional conflict [5] if the final choice incorporates informed consideration of the effects of the available options and is congruent with the woman’s own values, beliefs and preferences. In pain relief in labour, if healthcare professionals and midwives can help women identify their expectations, improve their knowledge and ascertain their values, beliefs and preferences, then women will be better placed to make decisions they are happy with and feel confident making. It is not enough to merely offer an evidence-based information tool in the hope that informed choice and decision making will automatically follow [47]. It was not clear from women’s accounts whether values or expectations about pain relief are routinely sought, acknowledged or recorded by midwives; it was certainly not something that they raised during the interviews. If women’s values on this issue are to be sought routinely during antenatal appointments, midwives need to tailor the support and information for women in order to best support them during labour and birth. If women begin to discuss their values at an early stage of their pregnancy, this will also help midwives explore with women how their values and subsequent expectations are formed, and ensure that they are well informed and based on accurate, evidence-based information. Any decisions women make, especially those made during the antenatal period are done with a degree of uncertainty [48]; it is a situation under which definitive decisions cannot reliably be made [49]. This adds further weight to the suggestion that ultimate decisions on pain management ought to be left until labour has started, which would enable a woman to make decisions in response to the level of pain being experienced and the control she is able to have over both the pain and the situation around her. This model of decision making in labour would need to be supported by the midwives who are there to provide one to one care for a woman during labour – a level of support which is sometimes taken for granted in the NHS [50]. The effect of this one to one support of midwifery-led care should not be underestimated, since it has been shown to lead to a decrease in pain relief and an increase in spontaneous labour [51]. The quality of this support, and the women’s sense of involvement in decision making by care providers in labour, has been found by Hodnett et al. to matter to women more than the quality of pain relief [33].

Helping women identify and consolidate their values and consider their birth preferences could be supported by a modification of the current birth plan. The birth plan would need major revision, taking into account the results of this study and the lessons learnt from the field of shared decision making. As illustrated in the data, not all women find the current birth plan a useful tool, several women even stating that it undermines professionals. Due to the apparent negative image of the birth plan, the content and purpose would need to be reviewed and promoted to women and midwives as an aid to support their engagement in decision making in labour. A new birth plan for the NHS could be used to support assessment of knowledge, clarification of values and beliefs, identification of preferences and as a communication tool to support decision making. This new tool could consist of elements derived from the Ottawa Decision Support Framework (ODSF) [52], which incorporates knowledge assessment - checking the woman’s knowledge of the risks and benefits of options - identification of her values and current option preferences. These elements could also inform the content of antenatal education, decision aids and prenatal support groups. If such elements are incorporated into antenatal education and the revised birth plan, which could be completed and updated throughout pregnancy, is then shared with healthcare professionals at the time of delivery, it could go some way to ensuring that each woman’s values and preferences are considered during labour by those supporting her. This approach would enable a woman to get the support and the pain relief that is appropriate for her in the context of the actuality of experienced labour.

A major strength of this research is that it explores decision making in the specific context of the provision of maternity care and hence addresses the uncertainty that women face throughout pregnancy, given the difficulties in predicting the sort of labour they will have and how they will respond to pain. This research has been able to identify elements of support for decision making that may fit the needs of women preparing for labour in a way that previous research, conducted in other clinical settings, has been unable to do given the differences in the context of pregnancy and labour described above.

A further strength is that the qualitative methods used have enabled a detailed in-depth understanding of the subject area. Examination of shared decision making in childbirth is an emerging field of research and it is important to access and explore the views of women themselves. However, the women interviewed tended to focus on pharmacological methods of pain relief. The issue of natural methods of pain management was explored in the interviews, but the design of the study was such that the interviews focused primarily on issues raised by the women themselves.

The interviews were conducted by a lone researcher (JL), but analysed with a fellow researcher (CE). The interpretation of the interviews was shaped by the researcher’s own experiences and understanding of pain and its management in labour. JL is a non-clinical researcher, who had experienced labour and childbirth herself three times. When asked by the women whether or not she had any children, JL would offer this information. This information sharing allowed a “matching of oneself”, which Hallowell et al. proposed may help develop trust with participants [53]; in some interviews there was a noticeable difference in the conversation once this disclosure of shared identities had happened. One assumption made by the researcher was that the subtleties she understood of the differences of pain management and pain relief would be shared by the women. The focus of the discussion on pharmacological pain relief methods may point to the fact that the women themselves did not differentiate between management and relief of pain.

## Conclusion

The current approach of antenatal preparation in the NHS, asking women to make decisions antenatally for pain relief in labour, needs reviewing, based on the findings from this study. It would be more beneficial to concentrate efforts on better informing women and on engaging them in discussions around their values, expectations and preferences and how these affect each specific choice rather than expecting them to make firm decisions in advance of such an unpredictable event as labour.

## Competing interests

We do not have any interests to disclose.

## Authors’ information

Joanne Lally held a Doctoral Research Fellowship from the Medical Research Council to undertake a PhD study “Decision making in pregnancy and childbirth: Hopes, expectations and realities”.

Richard Thomson leads the Decision Making and Organisation of Care research programme in the Institute of Health and Society, Newcastle University and co-leads a programme on implementation of SDM (MAGIC – Making Good Decisions in Collaboration) with Glyn Elwyn in Cardiff, supported by the Health Foundation.

Sheila MacPhail is a Consultant Obstetrician in The Newcastle upon Tyne Hospitals NHS Foundation Trust and has a research interest in decision making in pregnancy and childbirth.

Catherine Exley is Senior lecturer in Medical Sociology, Institute of Health and Society, Newcastle University.

## Authors’ contributions

JL designed the study, conducted the interviews, and was primarily responsible for data analysis and interpretation. RT and SM co-designed the study, supervised JL in conducting this study, contributed to the data interpretation and manuscript preparation. CE supervised the study, supported data analysis and assisted with data interpretation, and contributed to the first draft of the manuscript. All authors read and approved the final manuscript.

## Pre-publication history

The pre-publication history for this paper can be accessed here:

http://www.biomedcentral.com/1471-2393/14/6/prepub
